# Clinical-radiomic analysis for non-invasive prediction of liver steatosis on non-contrast CT: A pilot study

**DOI:** 10.3389/fgene.2023.1071085

**Published:** 2023-03-20

**Authors:** Shengnan Tang, Jin Wu, Shanshan Xu, Qi Li, Jian He

**Affiliations:** ^1^ Department of Nuclear Medicine, Nanjing Drum Tower Hospital, The Affiliated Hospital of Nanjing University Medical School, Nanjing, China; ^2^ Department of Pathology, Nanjing Drum Tower Hospital, The Affiliated Hospital of Nanjing University Medical School, Nanjing, China

**Keywords:** NAFLD, liver steatosis, non-contrast computed tomography, radiomics features, nomogram

## Abstract

**Purpose:** Our aim is to build and validate a clinical-radiomic model for non-invasive liver steatosis prediction based on non-contrast computed tomography (CT).

**Methods:** We retrospectively reviewed 342 patients with suspected NAFLD diagnoses between January 2019 and July 2020 who underwent non-contrast CT and liver biopsy. Radiomics features from hepatic and splenic regions-of-interests (ROIs) were extracted based on abdominal non-contrast CT imaging. The radiomics signature was constructed based on reproducible features by adopting the least absolute shrinkage and selection operator (LASSO) regression. Then, multivariate logistic regression analysis was applied to develop a combined clinical-radiomic nomogram integrating radiomics signature with several independent clinical predictors in a training cohort of 124 patients between January 2019 and December 2019. The performance of models was determined by the area under the receiver operating characteristic curves and calibration curves. We conducted an internal validation during 103 consecutive patients between January 2020 and July 2020.

**Results:** The radiomics signature was composed of four steatosis-related features and positively correlated with pathologic liver steatosis grade (*p* < 0.01). In both subgroups (Group One, none *vs*. steatosis; Group Two, none/mild *vs*. moderate/severe steatosis), the clinical-radiomic model performed best within the validation cohort with an AUC of 0.734 and 0.930, respectively. The calibration curve confirmed the concordance of excellent models.

**Conclusion:** We developed a robust clinical-radiomic model for accurate liver steatosis stage prediction in a non-invasive way, which may improve the clinical decision-making ability.

## Introduction

Non-alcoholic fatty liver disease (NAFLD) refers to a circumstance characterized by overmuch hepatic fat accumulation, and its prevalence is growing at an incredible rate around the world due to the obesity epidemic (Allen et al., 2018). As a hepatic presentation of the metabolic syndrome, NAFLD is often accompanied by obesity, dyslipidemia or insulin resistance (Loomba and Sanyal, 2013). In recent years, NAFLD has become the most common cause of serum aminotransferase abnormalities as well as a chronic liver disease within western countries, and the more severe liver steatosis is proven to has a closer relationship with non-alcoholic steatohepatitis (NASH) and the increased risk of liver cancer (Chalasani et al., 2008; White et al., 2012; Piscaglia et al., 2016; Leoni et al., 2018; Wang et al., 2022a). Due to its high incidence and potential risks, it can be predicted that early detection of high-risk individuals and timely intervention of NAFLD will become one of the most vital tasks in the field of liver disease in the coming decades (Hu et al., 2020).

NAFLD is a spectrum characterized by pathological features of diffuse hepatic steatosis and triglyceride accumulation (Fan et al., 2019). Although biopsy is widely recognized as the standard method for evaluating diffuse liver diseases, there are several shortcomings, including interobserver variability, costs, tissue sampling errors and complications such as pain, bleeding, infection or even mortality (Samir et al., 2015; Tang et al., 2015; Homayounieh et al., 2020). Simple and cost-effective diagnostic modalities and non-invasive ways to minimize the need for a liver biopsy are now recommended in both European and American clinical guidelines of NAFLD (Marchesini et al., 2016; Chalasani et al., 2018). For this, various non-invasive methods have been developed these years, including several diagnostic panels of clinical and serological evaluations, such as hepatic steatosis index (HIS), fatty liver index (FLI) and NAFLD liver fat score (NAFLD-LFS), etc, (Bedogni et al., 2006; Lee et al., 2010; Fedchuk et al., 2014). However, none of them was able to distinguish between moderate and severe stages of hepatic steatosis.

Nowadays, imaging modalities behave well with favourable capacity in diagnosing hepatic steatosis, including US, MRI, CT, etc, (Marchesini et al., 2016). Compared to CT and MRI, US remains the first-line imaging tool for NAFLD diagnosis due to the advantage of simplicity, reproductivity and inexpensiveness (Monica et al., 2021). However, it has insufficient sensitivity and even fails to detect steatosis when <20% or facing high body mass index (BMI) individuals (Ryan et al., 2002; Saadeh et al., 2002; Fishbein et al., 2005). Among the exiting approaches, ^1^H-MRS is the only way can obtain a quantitative liver fat estimation, but it is costly and not suitable for routine clinical application (Musso et al., 2010). CT can diagnose moderate and severe steatosis robustly and offer extra hepatobiliary information. Therefore, it is still the most frequently used method for hepatic steatosis assessment. In addition, many patients only accept non-contrast CT in China due to the low radiation exposure and reasonable cost-effectiveness (Marrero et al., 2018).

Radiomics is emerging as a promising field of imaging analysis technology, in which numerous quantitative features could be extracted from various invisible images (Gillies et al., 2016). We expect it a promising method to characterize the hepatic histological. Prior studies have indicated the potential of radiomics to detect NASH, fibrosis and cirrhosis within the field of benign liver disease (Naganawa et al., 2018; Wang et al., 2020). However, to our best knowledge, few studies focused on differentiating liver steatosis stage based on a combined clinical-radiomic analysis. We hypothesized that a clinical-radiomic model based on radiomics features extracted from the easily acquired non-contrast CT images combined with standard clinical parameters may improve steatosis grading accuracy. Therefore, our study aimed to develop and validate a clinical-radiomic model for staging liver steatosis making use of non-contrast CT.

## Materials and methods

### Patients

**FIGURE 1:**
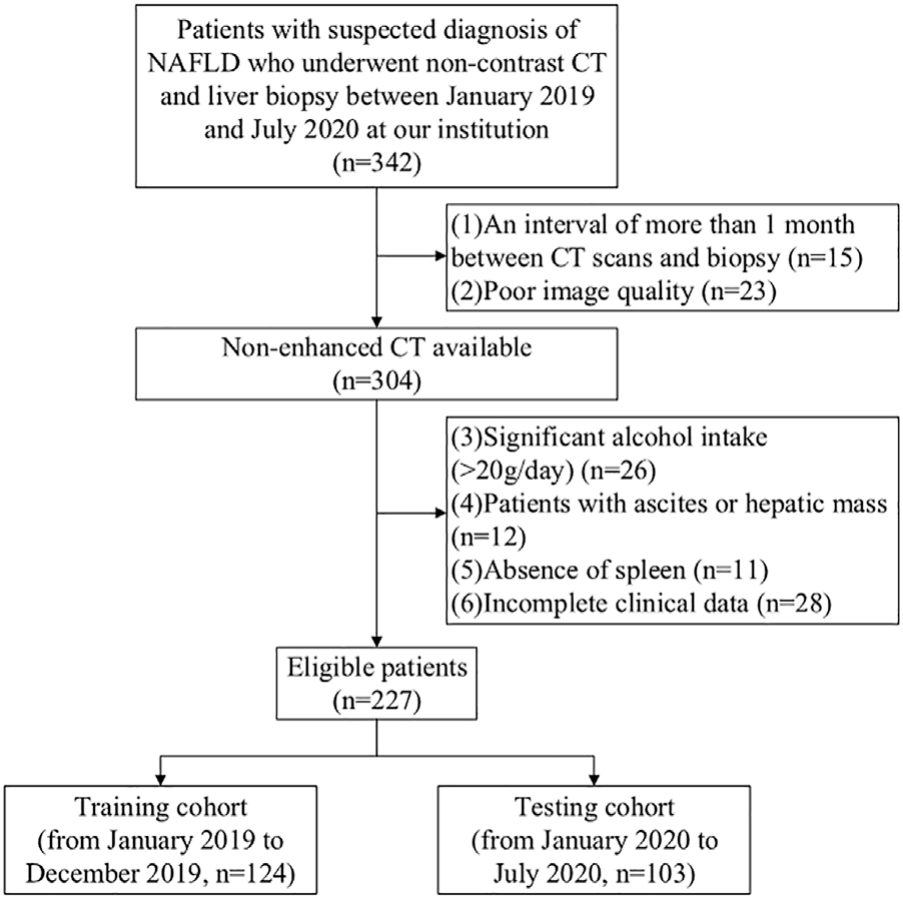
Patient selection flow diagram.

The Ethics Committee at our institution approved this study at our institute, and the informed consent was exempted due to the retrospective nature. We reviewed 342 patients who received liver biopsies to diagnose suspected NAFLD from January 2019 to December 2020 at our institution. All patients have done standardized non-contrast CT and serological tests during the treatment. The median interval between biopsy and imaging examination was 14 days. The exclusion criteria are shown in the patient selection flow diagram (Figure 1). Finally, a whole number of 227 patients were incorporated into this study, who were then assigned to two cohorts based on the admission time point: 1) A training cohort of 124 patients between January 2019 and December 2019 for model construction 2) A validation cohort of 103 patients between January 2020 and December 2020 for internal validation.

All clinical characteristics and laboratory results were acquired from medical records. Clinical data included age, gender, body mass index (BMI), hypertension, diabetes, smoking, blood routine tests (red blood cell [RBC], white blood cell [WBC] and platelet [PLT]), liver function examinations (alanine aminotransferase [ALT], aspartate aminotransferase [AST], y-glutamic transpeptidase [GGT], total bilirubin and albumin) and lipid metabolism tests (cholesterol and triglycerides). Two fibrosis-related indexes (APRI, aminotransferase-to platelet ratio index; FIB-4, fibrosis-4 index) were also calculated from data obtained from the laboratory tests. APRI was calculated as (aspirate trans inane [international units/liter]/upper normal limit × 100/platelet counts [×109/liter]) and fibrosis-4 index as (age[years] × aspirate transaminase [international units/liter])/(platelet counts [×109/liter] × alanine aminotransferase [international units/liter]1/2) (Bedogni et al., 2006; Lee et al., 2010).

### Liver biopsy

All patients underwent percutaneous biopsy in the right lobe of the liver under ultrasound guidance by experienced ultrasound radiologists. Samples were formalin-fixed and paraffin-embedded for further analysis. Two pathologists (with more than 6 years of experience in liver pathology) histologically analyzed the liver biopsy specimens in consensus, who were blinded to the clinical information and study design. Liver steatosis was graded by the liver parenchyma involvement of steatosis as S0 (none, <5%), S1 (mild, 5%–33%), S2 (moderate, 33%–66%) and severe (>66%). Hepatic fibrosis and inflammation activity assessment were also recorded. All the grading and staging is according to the standard Kleiner Classification (Kleiner et al., 2005).

### CT image acquisition and attenuation measurements

All participants underwent CT scan in supine position using multidetector spiral CT scanners (Lightspeed, VCT, or GE Healthcare, US). The unified CT parameters were as follows: tube voltage, 120 kVp; tube current, 250-350 mA; collimating slice thickness, 5 mm; reconstruction slice thickness, 1.25 mm; slice interval 1.25 mm, rotation time 0.6 s, helical pitch 1.375x, the field of view between 35 and 40 cm, matrix 512 × 512. A standard reconstruction algorithm was employed.

**FIGURE 2:**
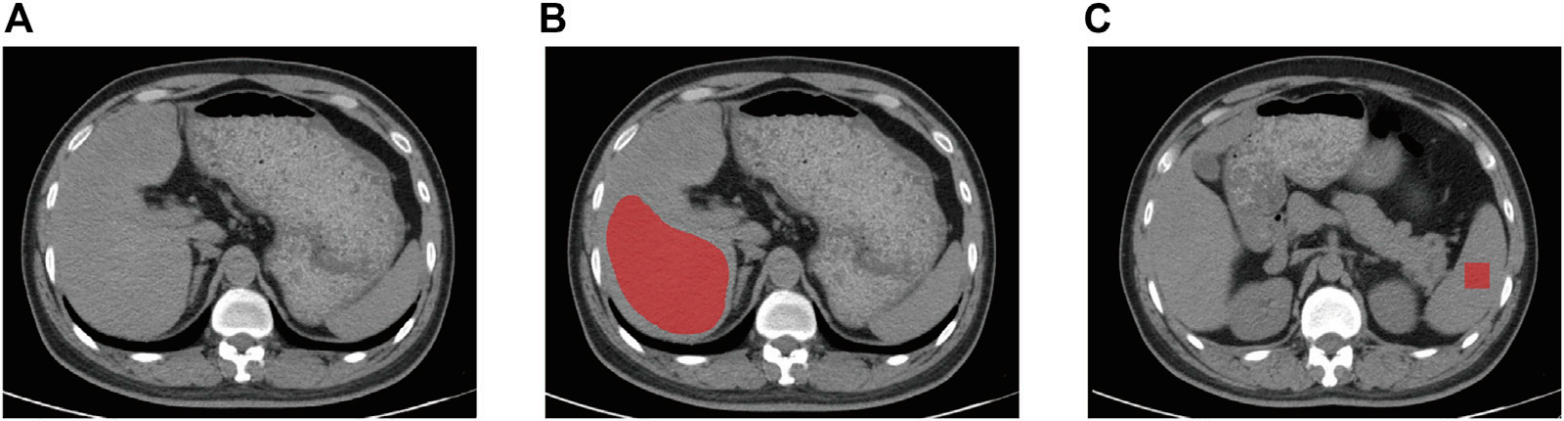
Non-contrast CT image of a 42-year-old man with mild liver steatosis. The ROI of liver **(A)** and spleen **(B)** is indicated by the red area. The ROI of liver was delineated along the margin of the right hepatic lobe at the level of right portal vein by avoiding large hepatic vessels and the ROI for spleen is 4 cm^2^ square of two contiguous sections. CT, computed tomography, ROI, region of interest.

Two imaging physicians with 5 and 7 years of abdominal imaging experience retrospectively assessed all non-contract CT scans, and all observers were blinded to the clinical or pathological details. Any disagreement was solved through regular discussions. After some substandard images had been excluded, all the CT images were assigned to them to measure hepatic and splenic attenuation. Two liver-related attenuation indices were attained for each enrolled image using the non-contrast CT images, where L represents hepatic attenuation and S represents splenic attenuation (Park et al., 2006; Sang et al., 2007). The first index, the liver-to-spleen attenuation ratio (CT_L/S_), was calculated as L/S, the second index, the difference between the hepatic and splenic attenuation (CT_L-S_) was obtained by L—S. Hepatic attenuation was calculated as three ROIs’ mean Hounsfield units, and each ROI consisted of a 2-cm diameter circle. Lands were settled within three adjacent locations in the right liver lobe, where they matched the biopsy sites as accurately as possible. Splenic attenuation was acquired through the average of three Hounsfield units at three different sections of the spleen (upper third, middle and lower third). A circular ROI with 2 cm in diameter was used for each measurement (Figure 2).

### Region-of-interest segmentation and radiomics feature extraction

All the image data were obtained from PACS (Picture Archiving and Communication system) and imported into ITK-SNAP software (version 3.8.0; http://www.itksnap.org). Two radiology residents, both with over 5 years of experience within imaging diagnosis and processing, were involved in the manual segmentation of the liver and spleen of all patients. ROIs for the liver were delineated along the margin of the right hepatic lobe at the level of the right portal vein by avoiding large hepatic vessels of two contiguous sections. Two 2 × 2 cm rectangular ROIs were placed at the middle of the spleen on two contiguous slices.

We applied an open-source R tool package called Pyradiomics (http://www.radiomics.io/pyradiomics.html) for image preprocessing and feature extraction. The voxel spacing was standardized with 1 × 1 × 1 mm, and voxel intensity values were discretized with a bin width of 25 HU to reduce the image noise interference and normalize intensities. Eight hundred thirty-seven radiomics features were composed of 18 first-order features, 75 textual features and 744 wavelet-based transformations. Features related to shape and size were excluded because they mainly reflected the manually delineated ROIs. To evaluate the reliability of each radiomics feature, we used the inter-and intraobserver intraclass correlation coefficient (ICC) based on 30 randomly picked subjects. First, Reader 1 conducted the ROI segmentation twice in the one-month interval. Then, Reader two performed all the ROI segmentation independently to calculate inter-and intraobserver ICC. We considered an ICC of 0.8 as the prescribed minimum of this study.

### Feature selection and radiomic model establishment in the training cohort

Radiomics features went through a selection process of two steps to overcome the shortcomings of traditional logistic regression methods. Firstly, features with high reproducibility (ICC >0.8 in intra- and interobserver settings) were retained for further analysis. Secondly, LASSO regression analysis with penalty parameter tuning conducted by 10-fold cross-validation was used to select the training cohort’s steatosis-related features with non-zero coefficient. We selected LASSO because of its interpretation advantage and its excellent performance in multiple studies about radiomics (Ji et al., 2019; Wang et al., 2022b; Wang et al., 2022c). We adopted Support vector machine (SVM) based on selected radiomics feature for model training on R software (version 3.6.1, http://www.r-project.org). SVM is a type of machine learning algorithm which is widely used to implement classification tasks (Hodgdon et al., 2015; Moller et al., 2016; Ta et al., 2018). The radiomics signature for the prediction of liver steatosis was constructed using SVM algorithm as a classifier to distinguish the steatosis stage. The type of SVM was “eps-classification”, of which the kernel function was radial basis. We divided our data into two groups to distinguish the liver steatosis stage. We divided our data into two groups to distinguish the liver steatosis stage. Group One recognizes the existence of NAFLD between stage S0 and S1-S3; Group Two distinguishes the mild and moderate-severe liver steatosis between stage S0-S1 and S2-S3. Both models of the two groups were established as binary classification models instead of multinomial ones because the latter one is complicated and generated multiple possible values for different steatosis stages. Compared to the complex model, a binary model is composed of a more straightforward equation and returns a single probability value, contributing to ease of interpretability (Park et al., 2019).

### Clinical factors selection

We performed two steps to select steatosis-related clinical factors. First, we applied Spearman correlation analysis for preliminary screening of all the parameters with significant correlation (*p* < 0.05). Next, forward conditional logistic multivariable analysis (input and output *p*-value: 0.05 and 0.1, respectively) was conducted for further selection. The cutoff value of each independent factor was determined by receiver operating characteristic (ROC) analysis (maximizing the Youden index).

### Development and validation of prediction models

We applied multivariate logistic regression analysis to develop models for liver steatosis prediction in the training cohort, including both clinical ones and clinical-radiomic ones. A nomogram was constructed in the training cohort to attain a more easily understood measure. Our models’ performance was then tested in the independent validation cohort by employing the formula and cutoff values acquired from the training cohort. Previous studies have developed some clinical models to detect steatosis. FLI (Fatty liver index) has been deemed as an accurate surrogate marker of hepatic steatosis in Asian and Western countries. We also calculated FLI using our clinical parameters for comparison to evaluate our models better, and an FLI >60 was considered as fatty liver (Bedogni et al., 2006).

### Statistical analysis

Categorical variables were compared with χ^2^ test or Fisher exact test. Continuous variables were compared with Mann-Whitney U test. The diagnostic performance of the established models was assessed through ROC curves and the area under the curve (AUC) values. A two-tailed *p*-value less than 0.05 was accepted statistically significant. All statistical analyses were performed using R software (version 3.6.1, http://www.rproject.org) or Statistical Product and Service Solutions (IBM SPSS, version 22.0; New York, NY).

## Results

### Patient characteristics

The baseline information of all participants was summarized in Table 1. No statistical differences were observed within clinical-radiological-pathological parameters between the training (n = 124) and validation cohorts (n = 103). The rates of patients with NAFLD are 38.7% (48 in 124) and 29.1% (30 in 103), and the rates of patients with moderate-severe NAFLD is 10.5% (13 in 124) and 8.7% (9 in 103) in the training and validation cohorts, respectively, while no difference was found between the two cohorts (c2, *p* = .450).

**TABLE 1:** Baseline characteristics.

Parameter	Training (*n*=124)	Validation (*n*=103)	*P* value
Age^*^ (years)	58 (52.75-65.25)	57 (50-65)	.224
Gender (male/female, n)	98/26	80/23	.804
BMI^*^ (kg/m^2^)	23.2 (21.07-25.00)	24.06 (21.64-25.81)	.244
Hypertension	38 (30.6)	31 (30.1)	.929
Diabetes	21 (16.9)	21 (20.4)	.505
Smoker	21 (16.9)	27 (26.2)	.088
CT attenuation^*^(HU)			
CT_L_	59.65 (53.89-63.21)	59 (54.34-65)	.277
CT_S_	50.45 (46.81-53)	46 (44.75-53)	.064
Laboratory findings^*^			
AST (IU/ml)	24.6 (18.08-36.83)	27.7 (18.15-48.90)	.073
ALT(IU/ml)	26.1 (19.2-34.5)	27.2 (21-37.65)	.239
AST/ALT	0.94 (0.91-1.06)	1.09 (1.02-2.32)	.514
GGT(IU/ml)	45.1 (26.58-69.4)	48.1 (26.05-89.55)	.303
Total bilirubin (ng/ml)	13.5 (10.08-17.4)	13.6 (9.35-18)	.865
Albumin (g/L)	40 (37.5-41.73)	39.6 (37.8-41.15)	.278
Cholesterol (mmol/L)	3.63 (3.04-4.15)	3.67 (3.36-4.17)	.211
Triglycerides (mmol/L)	0.94 (0.69-1.45)	0.98 (0.66-1.29)	.729
APRI	0.56 (0.35-0.86)	0.47 (0.32-1.03)	.745
FIB-4	2.65 (1.75-3.87)	3.11 (1.35-3.98)	.273
Histologic grade			
Steatosis			.450
S0 (none)	76 (61.3)	73 (70.9)	.130
S1 (mild)	35 (28.2)	21 (20.4)	.173
S2 (moderate)	10 (8.1)	6 (5.8)	.512
S3 (severe)	3 (2.4)	3 (2.9)	.818
Fibrosis (F0/F1/F2/F3/F4)	12/26/17/32/37	9/14/19/27/34	.597
Activity (A0/A1/A2/A3/A4)	0/50/58/14/2	3/36/47/16/1	.287

Data are the number of patients; data in parentheses are percentages unless otherwise indicated.

BMI, body mass index; HU, hounsfield unit; ALT, alanine aminotransferase; AST, aspartate aminotransferase, GGT = γ-glutamyl transpeptadase, APRI, aspartate aminotransferase-to-platelet ratio, FIB-4, fibrosis-4 index.

*Data are medians, with interquartile range in parentheses.

### Feature selection and radiomics signature establishment

**FIGURE 3:**
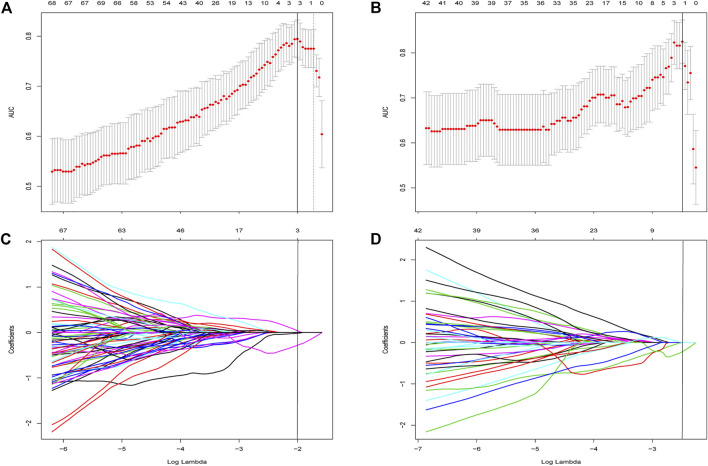
Feature selection using the least absolute shrinkage and selection operator (LASSO) regression within features extracted from liver. **(A)** Selection of tuning parameter (λ) was determined by the LASSO model using 10-fold cross validation *via* minimum criteria. The AUC curve was plotted *versus* log (λ). Dotted vertical lines were drawn at the optimal values by using the minimum criteria and the 1 standard error of the minimum criteria (the 1—Standard error criteria). The optimal λ value of 0.135 with log (λ) of −2 was chosen in Group One. **(B)** Feature selection in Group 2 and the optimal λ value of 0.083 with log (λ) of −2.49 was chosen. LASSO coefficient profiles of the 158 initially selected features in Group One **(C)** and Group Two **(D)**. A vertical line was placed at the optimal λ value, which resulted in three features with non-zero coefficients in both subgroups.

A total of 837 radiomics features were extracted from non-contrast CT, 158 and 89 (based on liver and spleen in two models, respectively) most stable features of liver and spleen that with high reproducibility were picked for further processing in both two groups. Four stable steatosis-related features with non-zero coefficients screened from the lasso regression model were selected based on the training cohort in the two radiomic models, respectively (both were three from the liver and one from the spleen). (hepatic features in Figure 3, spleen features in Supplementary Figure S1). The detailed description of radiomics features can be found in Supplementary Materials.

**FIGURE 4:**
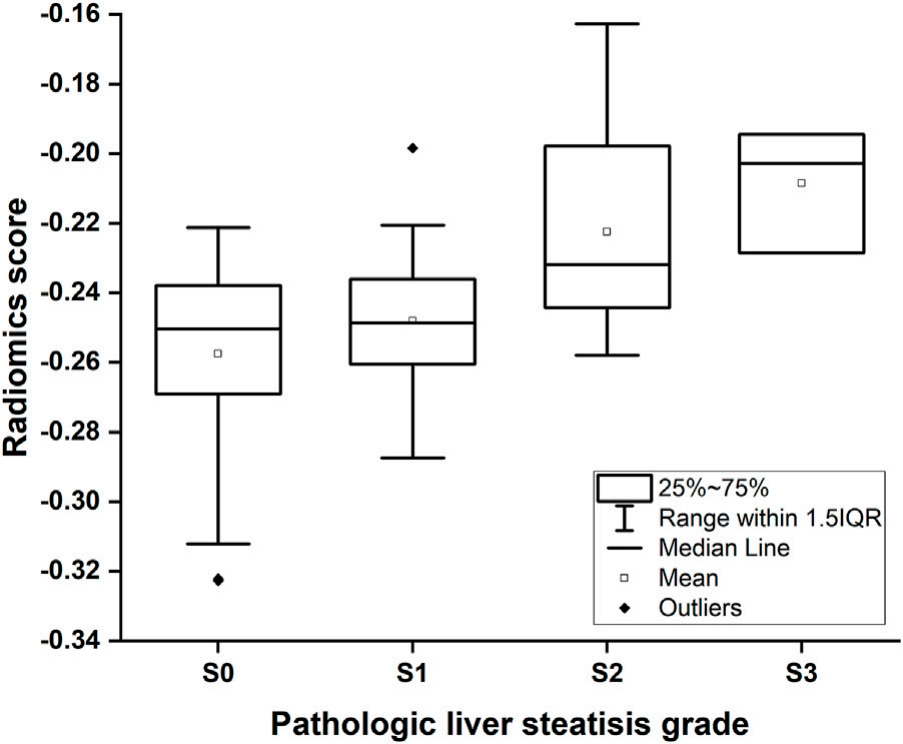
Box-and-whisker plot of the radiomics score for each pathologic liver steatosis stage calculated from the radiomics model in the training cohort. The median values of the radiomics score for stage S0, S1, S2 and S3 were −0.250, −0.248, −0.232, and −0.203, respectively.

The radiomics signature was established making use of the SVM algorithm. The radiomics score indicated a positive correlation with pathologic liver steatosis grade (*p* < .01, Figure 4). In Group One, we found a difference in radiomics score between patients with and those without steatosis in the training cohort (mean, −0.591 *vs*. −0.191, *p* < .001), and then confirmed in the validation cohort (mean, −0.526 *vs*. −0.426, *p* < .001). In Group Two, the radiomics score was compared between patients with none-mild steatosis and patients with moderate-severe steatosis in the training cohort (mean, −0.255 *vs*. −0.219, *p* < .001), and then confirmed in the validation cohort (mean, −0.236 *vs*. −0.101, *p* = .001). The radiomics signature showed favourable classification ability with an AUC of 0.808, 0.791 (training cohort and validation cohort) in Group one and 0.835, 0.851 (training cohort and validation cohort) in Group two.

### Steatosis-related clinical factors

In the trainingcohort, BMI, ALT/AST, albumin, triglycerides, CT_L-S_ and CT_L/S_ were found to be related to liver steatosis in Group one (*p* < .05, Spearman correlation analysis). Using the same method, BMI, ALT/AST, albumin, cholesterol, triglycerides, CT_L-S_ and CT_L/S_ were found to be significantly relevant to moderate-severe steatosis in Group two. Next, the multivariable conditional logistic regression analysis identified BMI, triglycerides, CT_L-S_ as independent steatosis predictors in both two of the models (Table 2). Cutoff values of BMI, triglycerides, CT_L-S_ were 24.82 kg/m2, 0.77 mmol/L, 7.2 HU in Group one 1 and 25.5 kg/m2, 1.17 mmol/L, 5.42 HU in Group two, respectively.

**TABLE 2:** Clinical characteristics selection in the training cohort related to liver steatosis.

	Model 1(S0 vs S1-S3)				Model 2 (S0-S1 vs S2-S3)			
	Spearman correlation analysis		Multivariable analysis		Spearman correlation analysis		Multivariable analysis	
Variables	*r* ^*2*^ value	*P* value	*b* coefficient	*P* value	*r* ^*2*^ value	*P* value	*b* coefficient	*P* value
Age	0.001	.795	NA	NA	0.001	.700	NA	NA
Sex	0.000	.977	NA	NA	0.002	.605	NA	NA
BMI	0.120	<.001	0.206	.025	0.078	.002	0.269	.048
Hypertension	0.014	.191	NA	NA	0.000	.992	NA	NA
Diabetes	0.001	.362	NA	NA	0.003	.536	NA	NA
Smoker	0.000	.950	NA	NA	0.000	.876	NA	NA
ALT	0.017	.149	NA	NA	0.004	.460	NA	NA
AST	0.000	.848	NA	NA	0.003	.562	NA	NA
ALT/AST	0.057	.008	NA	NA	0.033	.045	NA	NA
GGT	0.010	.268	NA	NA	0.009	.298	NA	NA
Total bilirubin	0.003	.583	NA	NA	0.001	.694	NA	NA
Albumin	0.076	.002	NA	NA	0.073	.002	NA	NA
Cholesterol	0.025	.081	NA	NA	0.052	.011	NA	NA
Triglycerides	0.185	<.001	1.242	.006	0.141	.000	2.103	.018
APRI	0.009	.307	NA	NA	0.023	.092	NA	NA
FIB-4	0.029	.059	NA	NA	0.028	.064	NA	NA
CT_L-S_	0.211	<.001	-0.164	<.001	0.200	<.001	-0.297	<.001
CT_L/S_	0.205	<.001	NA	NA	0.203	<.001	NA	NA

*b* coefficients are from multivariable logistic regression. Clinical variables found to be significantly related to liver steatosis through Spearman correlation analysis entered into forward conditional logistic multivariate analysis.

### Development, performance and validation of the established models

**FIGURE 5:**
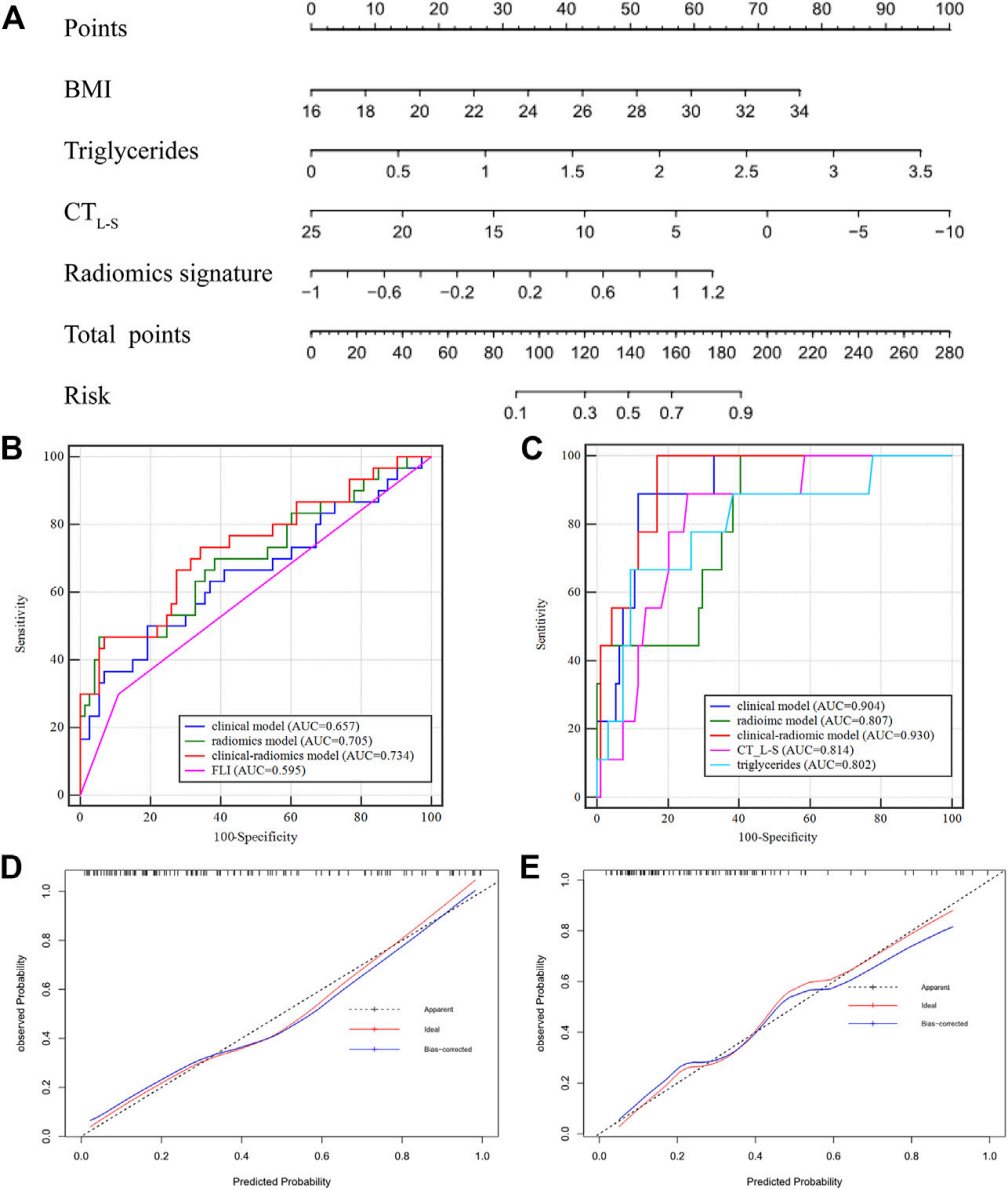
Nomogram developed with receiver operating characteristic curves and calibration curves. **(A)** A clinical-radiomic nomogram for distinguishing none from steatosis based on the training cohort, with BMI, triglycerides, CT_L-S_ and radiomics signature incorporated. **(B)** Comparison of ROC curve in the validation cohort of Group One (S0 vs. S1-S3) **(C)** comparison of ROC curve in the validation cohort of Group Two (S0-S1 *vs*. S2-S3). Calibration curves of the validation cohort in Group One **(D)** and Group Two **(E)**.

In Group one (none vs. steatosis), the formula of the clinical model was: Y1 (clinical) = 0.206 × BMI +1.242 × Triglycerides—0.164 × CT_L-S_—5.494 and the formula of the combined clinical-radiomic model was Y1 (clinical-radiomic) = 0.189 × BMI +1.215 × Triglycerides—0.127 × CT_L-S_ + 1.273 × Radiomics Signature—4.771. As shown in Figure 5A, a combined clinical-radiomic model that integrated the radiomics signature, triglycerides, BMI and CT_L-S_ was developed and presented as a nomogram. ROC analysis compared the discrimination capacities of the clinical-radiomic nomogram to those of the clinical model, radiomic model, CT_L-S_ and FLI in Figure 5B. As summarized in Table 3, the combined clinical-radiomic model performed best with an AUC value of 0.734 (95%CI: 0.638, 0.816; *p* < .001), which was higher than that of the clinical model (AUC, 0.657 [95%CI: 0.557, 0.748]; *p* = .015), the radiomic model (AUC, 0.705 [95%CI: 0.607, 0.790]; *p* < .001) or FLI (AUC, 0.595 [95%CI: 0.494, 0.691]; *p* = .04) and statistically significant (*p* < .05). The calibration curve was plotted to assess the nomogram, which showed great consistency between predicted and actual in the validation cohort (Figure 5D). The Hosmer-Lemeshow test yielded a *p*-value of 0.318, indicating no departure from the good fit.

**TABLE 3:** Diagnostic performance of all methods for predicting liver cirrhosis in the validation cohort.

	Group One (none vs. steatosis)		Group Two (none/mild vs. moderate/severe steatosis)	
Methods	AUROC (95%CI)	Cutoff value	AUROC (95%CI)	Cutoff value
Integrated model	0.734 (0.638-0.816)	0.20	0.930 (0.863-0.971)	-0.32
Radiomics model	0.705 (0.607-0.790)	0.48	0.807 (0.718-0.878)	-0.22
Clinical model	0.657 (0.557-0.748)	-0.10	0.904 (0.830-0.953)	-2.88
FLI	0.595 (0.494-0.691)	*NA*	*NA*	*NA*
CT_L/S_	0.600 (0.499-0.695)	7.67	0.814 (0.726-0.884)	7.67
Triglycerides	0.681 (0.582-0.769)	0.96	0.802 (0.712-0.874)	1.53
Comparison of AUROC (Delong test)				
Integrated model vs. Clinical model	*P* = .005		*P* =.397	
Integrated model vs. Radiomics model	*P* = .035		*P* =.016	
Integrated model vs. FLI	*P* = .026		*NA*	

AUROC, area under the receiver operating characteristic; FLI, fatty liver index.

In Group two (none-mild vs. moderate-severe steatosis), the formula of the clinical model was: Y2 (clinical) = 0.269 × BMI +2.103 × Triglycerides—0.297 × CT_L-S_—10.273 and the formula of the combined clinical-radiomic model was Y2 (clinical-radiomic) = 2 .503 × Triglycerides—0.369 × CT_L-S_ + 110.818 × Radiomics Signature +22.013. ROC analysis compared the discrimination ability of the clinical-radiomic nomogram to those of the clinical model, the radiomic model and two independent factors (CT_L-S_ and triglycerides) in Figure 5C. As summarized in Table 3, similarly, the combined clinical-radiomic model showed the best result with an AUC value of 0.930 (95%CI: 0.863, 0.971; *p* < .001), higher than that of the clinical model (AUC, 0.904 [95%CI: 0.830, 0.953]; *p* < .001), the radiomic model (AUC, 0.807 [95%: 0.718, 0.878]; *p* < .001), CT_L-S_ (AUC, 0.814 [95%: 0.726, 0.884]; *p* < .001), Triglycerides (AUC, 0.802 [95%: 0.712, 0.874]; *p* < .001). We found that there was a statistical difference between the combined clinical-radiomic model and the radiomics model. However, though the results of the combined clinical-radiomic model are better than the clinical model, no statistical difference was observed between them. The favourble calibration was also confirmed in the validation cohort by the calibration curve (Figure 5E) and the Hosmer-Lemeshow test (*p* = 0.481).

## Discussion

In this retrospective study, we attempted to use clinical-radiomic analysis for non-invasive prediction of liver steatosis severity by using non-contrast CT based on 342 patients with the suspected diagnosis of NAFLD. The radiomics signature was comprised of four stable radiomics features and had excellent discrimination ability in two subgroups. For ease of clinical utility, we developed a clinical-radiomic nomogram which integrated radiomics signature with several clinical indicators, including BMI, triglycerides and CT_L-S_, and achieved satisfactory results.

During radiomic features selection and radiomics signature establishment, Lasso regression and SVM algorithm were applied. We chose the LASSO model owing to its model interpretability benefit and its excellent performance in multiple radiomics-related research (Ji et al., 2019; Wang et al., 2022b; Wang et al., 2022c). The advantage of the SVM is that an SVM classifier relies merely on the support vectors, and the classifier function is not affected by the whole dataset. The SVM’s another trait is the possibility to tackle a large number of features thanks to kernel functions’ exploitation (Kim et al., 2011). Among the four radiomics features finally selected, three are from the liver and one is from the spleen, which may reflect the calibration effect of the image of the spleen on liver steatosis.

We included several clinical parameters during our model development which were selected by using univariate correlation analysis and multivariable analysis. BMI, triglycerides and CT values have also been reported in previous steatosis-related studies. Although about one in six of the NAFLD patients has a normal BMI (“lean NAFLD”), obesity relates to an increased risk of NAFLD and the risk increases with increasing BMI (Alferink et al., 2019; Pang et al., 2019). Hypertriglyceridemia is known as a risk factor for developing newly NAFLD and has been included in several clinical models (Fedchuk et al., 2014). CT values may represent as the earliest indicator of hepatic steatosis, which includes many calculation forms including CT values of liver alone, CT liver/spleen ratio (CT_L/S_), blood-free hepatic parenchymal attenuation (CT_L/P_), etc., we included two of the most commonly applied indices into our statistical analysis (Park et al., 2006; Kodama et al., 2007; Sang et al., 2007).

Within the comparison between the combined model and other models, In Group One (none vs. steatosis), we established radiomic model, clinical model and combined clinical-radiomic model, and we found that the integrated model performed best followed by the radiomic model and clinical model. Statistically significant differences have been validated by the Delong test, which illustrated that the radiomics signature played a vital role in distinguishing stage S0 from S1-S3. In Group Two (none/mild *vs*. moderate/severe steatosis), we also established models using the same method. The combined model still performed the best, but the difference did not achieve a statistical significance. Several possible reasons may help explain this. First is the small sample size of our internal validation cohorts. Second, an unbalanced proportion may account for the statistical results. Last but not least, patients with severe fatty liver may show prominent clinical characteristics and imaging features that were visible with naked eyes. Therefore, although the comprehensive model performs slightly better than the clinical model, it does not show statistical significance.

Our study also had several limitations. First, there might exist inevitable selection bias owing to the retrospective nature of our study. There were a larger number of patients in the lower steatosis stage, and the uneven population distribution may overestimate diagnosis performance. However, we found no significant difference of clinical-radiological-pathological characteristics between the training and validation cohorts. Second, our model was developed and validated on the basis of a single-centre database and experience where multi-institutional validation is required in the future. Third, although we tried to avoid visible hepatic vessels by manually delineating ROIs in the right lobe of liver, it can be challenging for the naked eyes to identify vessels under the background of the fatty liver because of the similar CT value. However, the vessel area has less influence due to the small area in the peripheral zone of liver. Forth, we use ROI instead of VOI for feature selection which may result in loss of partial image information. But if the 2D method can achieve similar results compared with the 3D method, it can greatly simplify the application. After all, manually delineation of all the liver and spleen is relatively labor intensive, and it has been proved and practiced in previous studies (Kim et al., 2011; Meng et al., 2021).

In conclusion, we established and validated a non-invasive and convenient clinical-radiomics model to predict the steatosis severity based on traditional non-contrast CT. Open-source software and a standard toolkit were used to delineate ROIs and extracted radiomics features, and four stable steatosis-related radiomics features were chosen from 837 features and establish models with the SVM algorithm. Then, we further included the clinical factors and developed the integrated model to predict the steatosis stage accurately. The clinical-radiomic model can early detect the high-risk population and may help clinical decision-making.

## Data Availability

The raw data supporting the conclusion of this article will be made available by the authors, without undue reservation.
