# Supplementary material for: Clinical-radiomic analysis for non-invasive prediction of liver steatosis on non-contrast CT: A pilot study
Source: Front Genet. 2023 Mar 20;14:1071085. doi: 10.3389/fgene.2023.1071085 (PMC10069650; doi:10.3389/fgene.2023.1071085)

Supplementary materials

1. Radiomics features description

Both two model including 4 stable radiomics features (three from liver and one from spleen), but there are slight differences between them.

In Group 1, there are three features from liver including two frist-order features (energy and energy_H) and one textual feature (large area low gray level emphasis) and one feature from spleen (textual feature: gray level non-uniformity).

In Group 2, there are three features from liver including two frist-order features (energy and minimum) and one textual feature (large area low gray level emphasis) and one feature from spleen (textual feature: gray level non-uniformity).

Interpretation of the features:

（1）Energy: Energy is a measure of the magnitude of voxel values in an image. A larger values implies a greater sum of the squares of these values.

（2）Energy_H: Energy_H is a wavelet transformed feature from Energy.

（3）Large area low gray level emphasis (LALGLE): LALGLE measures the proportion in the image of the joint distribution of larger size zones with lower gray-level values.

（4）Gray level non-uniformity (GLN): GLN measures the variability of grey-level intensity values in the image, with a lower value indicating more homogeneity in intensity values.

2.Supplementary figure S1

Feature selection using the least absolute shrinkage and selection operator (LASSO) regression within features extracted from spleen. (a) Selection of tuning parameter () was determined by the LASSO model using 10-fold cross validation via minimum criteria. The AUC curve was plotted versus log (λ). Dotted vertical lines were drawn at the optimal values by using the minimum criteria and the 1 standard error of the minimum criteria (the 1 – standard error criteria). The optimal value of 0.119 with log (λ) of -2.15 was chosen in Group One. (b) Feature selection in Group 2 and the 461optimal value of 0.092 with log (λ) of -2.21 was chosen. LASSO coefficient profiles of the 158 initially selected features in Group One (c) and Group Two (d). A vertical line was placed at the optimal value, which resulted in three features with nonzero coefficients in both subgroups.


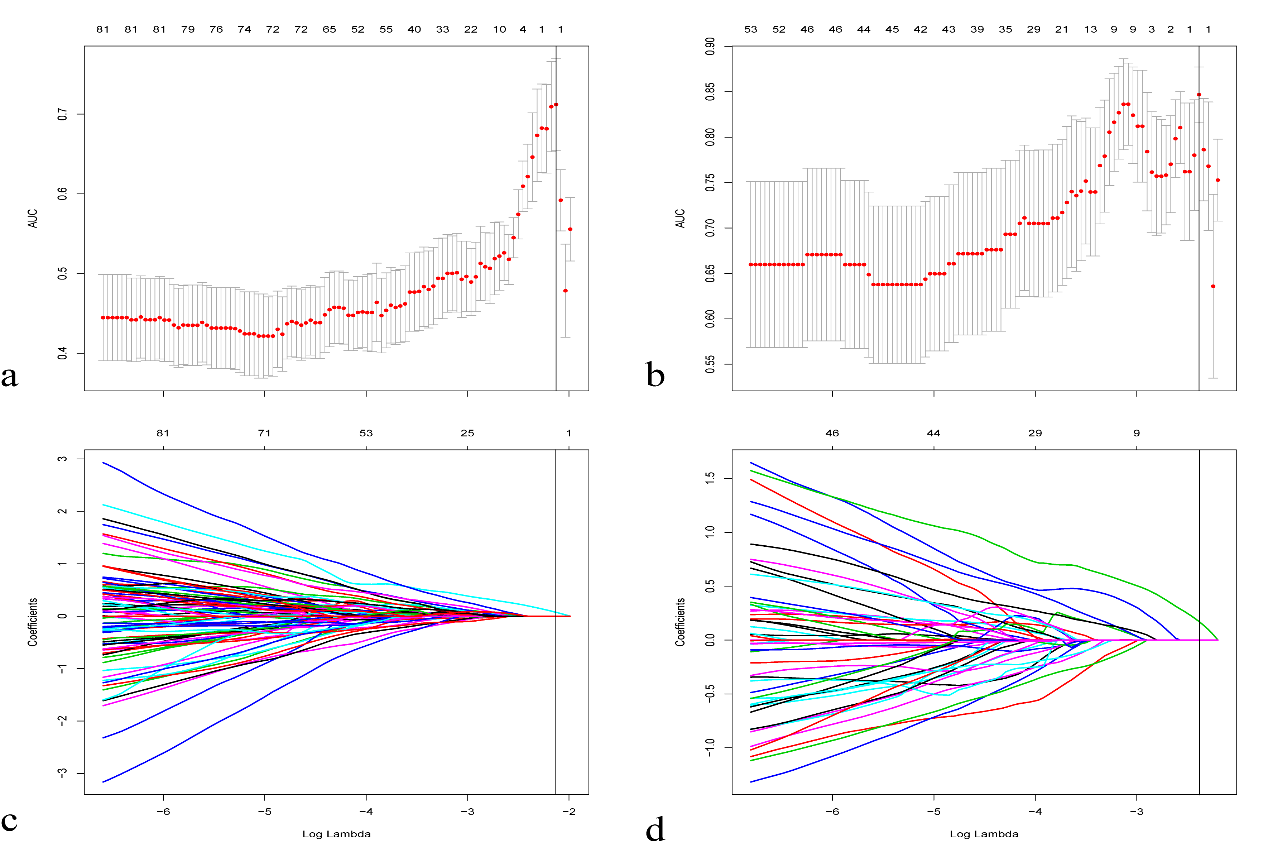

Supplement: Supplementary file 1 [file DataSheet1.docx]
